# A benchmarking study of father involvement in Australian child mental health services

**DOI:** 10.1371/journal.pone.0203113

**Published:** 2018-08-28

**Authors:** Mark R. Dadds, Daniel A. J. Collins, Frances L. Doyle, Lucy A. Tully, David J. Hawes, Rhoshel K. Lenroot, Vicki Anderson, Paul J. Frick, Caroline Moul, Eva R. Kimonis

**Affiliations:** 1 School of Psychology, University of Sydney, Sydney, New South Wales, Australia; 2 School of Psychiatry, University of New South Wales, Sydney, New South Wales, Australia; 3 Clinical Sciences, Murdoch Children’s Research Institute, Melbourne, Victoria, Australia; 4 School of Psychological Sciences & Department of Paediatrics, University of Melbourne, Melbourne, Victoria, Australia; 5 Learning Sciences Institute of Australia, Australian Catholic University, Brisbane, Queensland, Australia; 6 Department of Psychology, Louisiana State University, Baton Rouge, Louisiana, United States of America; 7 School of Psychology, University of New South Wales, Sydney, New South Wales, Australia; Arizona State University, UNITED STATES

## Abstract

Fathers are underrepresented in interventions focussing on child well-being, yet research suggests their involvement may be critical to enhancing intervention effectiveness. This study aimed to provide the first Australian benchmark of rates of father attendance across several child mental health services. Retrospective casefile reviews were conducted to obtain data on father and mother attendance at 10 Australian child mental health services. A total of 2128 casefile records were retrospectively examined to extract family-level data. The main outcome measures were rates of father and mother attendance at sessions involving parents, and rates of father- and mother-instigated referral to services. Across services, fathers attended on average 48.2% (range 39.7% to 72.0%) of total parent sessions, with an average of 68.4% (range 53.1% to 88.1%) of fathers attending at least one session. Mothers attended sessions at significantly higher rates; an average of 92.8% of total parent sessions and 96.9% attendance for at least one session. For self-referred families, on average 12.6% of referrals were from fathers, and 87.4% were from mothers. These results indicate that rates of father attendance at Australian child mental health services vary, but are significantly lower than attendance rates for mothers. This may compromise the quality and outcomes of child mental health services in Australia. Routine monitoring of rates of father attendance is needed, as are strategies to enhance father engagement.

## Introduction

Several review papers have concluded that fathers (i.e. male caregivers) are underrepresented in research and clinical interventions targeting child well-being (e.g., [1–6]). Parenting interventions typically aim to increase the quality and consistency of parenting and reduce coercive parent-child interactions; to date, research has shown better child outcomes are achieved when these interventions involve fathers as well as mothers [7, 8]. For example, in Lundahl and colleagues’ meta-analysis (*k* = 26), father involvement in parenting interventions was associated with improved parenting practices and positive changes in child behaviour in the short-term [7]. Other researchers have demonstrated long-term improvements in child outcomes when fathers are included in interventions along with mothers (e.g., [8]). Despite the importance of father participation to intervention outcomes, little research has documented the actual levels of father engagement in services for child well-being [4–6]. Therefore, it is critical to understand the current rate of father engagement in these interventions, in order to provide a benchmark against which to measure the effectiveness of future strategies for increasing father involvement.

Many practitioners who work with families perceive that there is a low rate of father attendance at services (e.g., [9, 10]). For example, in a survey of 210 Australian practitioners, only 17% reported that fathers *often* attended programs/services, whereas 29% reported that fathers *rarely* attended [9]. Despite these survey findings, there is little empirical data from child mental health services to document rates of father attendance. While a meta-analysis (*k* = 28) found 20% of participants in parenting interventions were fathers [4], this figure is based on the numbers enrolling in programs, not actual attendance rates. To our knowledge, there has been no systematic collation of data on fathers’ level of engagement in interventions for child well-being across a range of clinical settings. Thus, in this study, we aimed to provide a benchmark of father involvement using retrospective casefile data from several child mental health services around Australia.

A recent conceptual model, called CAPE, defines parental engagement across several stages from ‘Connecting’ (enrolment in a program), to ‘Attending’ (presence at sessions), ‘Participating’ (active participation during sessions), and finally ‘Enacting’ (implementing strategies) [11]. Session attendance is commonly used as a measurable and standardised proxy for engagement when examining parenting interventions (e.g., [12]). Therefore, in this study we focussed on father attendance (measured by presence at sessions) as the key measure of father engagement. We also considered father-instigated family referral as an additional indicator of father engagement, corresponding with the ‘Connecting’ stage of the CAPE model [11].

The primary aim of this study was to provide the first Australian benchmark of father attendance across several child mental health services, conducted via retrospective casefile review. The secondary aims of this study were to determine whether there was a significant difference between father and mother attendance rates, and to compare rates of self-referral between fathers and mothers.

## Methods

### Child mental health services

Fourteen Australian child mental health services were approached to participate in this study, which involved a retrospective casefile review of families who had previously attended their service. This method was selected as the services did not routinely collect prospective data on father attendance. Inclusion criteria were that services needed to work with children and families, involve parents directly in at least some aspects of the service provided (i.e. not exclusively provide child-only sessions), and be located in Australia. Half of the 14 services approached were part of a national network of child conduct disorder clinics that convened in 2013 and committed to participation in research. An additional seven services were approached to increase the number of services participating in the study. Four services (three in New South Wales [NSW], one in Australian Capital Territory [ACT]) that had planned to participate withdrew from the study. Three of these were non-government organisations and the other was a government child and family mental health service. The primary reason for withdrawal of these services was insufficient time/resources. The remaining 10 services (six in NSW, and one each in ACT, South Australia [SA], Western Australia [WA], and Victoria [VIC]) participated in this benchmarking study. The characteristics of participating services are provided in Table 1. Four were government child and family mental health services (2, 3, 4, and 5), three were services based at non-government or charitable organisations (6, 8 and 9), two were university-based psychology clinics (1 and 10), and one was a hospital-based psychology clinic (7). All services were offered free of charge with the exception of one university-based psychology clinic (1), which charged a nominal fee.

**Table 1:** Characteristics of participating services.

Service number	Service location	Service description	Offers sessions outside working hours?	Child age range
**1**	Canberra, ACT	University-based psychology clinic that provides assessment and treatment of children and adolescents with emotional and behavioural problems, with a greater focus on parent training for families with younger children. The first appointment is free; remaining sessions have a nominal fee.	No	3 to 17
**2**	Port Adelaide, SA	Free, community-based government child and adolescent mental health service. The majority of clients are young people and families who suffer from moderate to severe mental illness. Treatment types include general counselling, psychiatric assessments and longer term therapy.	No	Up to 16
**3**	Sydney, NSW	Free, specialist government mental health service providing comprehensive assessment, advocacy, counselling and other services for children who are experiencing emotional, behavioural or social difficulties, and their families. Interventions may be individual, family or group therapy based within an early intervention framework. The service has a family focus and offers a multidisciplinary approach aimed to prevent, identify, treat, and reduce the impact of these difficulties on children, young people and their families.	No	5 to 17
**4**	Perth, WA	Free, community-based government child and adolescent mental health service. Delivers Multisystemic Therapy (MST): an intensive home and community intervention for families with young people having conduct disorders or delinquent behaviour. This 4 to 6 month intervention teaches parents/caregivers problem-solving skills to manage their children’s behaviours, and improves communication between relevant parties (e.g. family and school). MST clinical staff are available on 24/7 call for all families throughout the intervention. The assigned clinician visits each family’s home 3 times per week during the intervention, and clinical visits are also typically made to the young person’s school and to other key community sites/stakeholders.	Yes	12 to 16 (10, 11 & 17 year olds also considered if severe cases)
**5**	Wollongong, NSW	Free, community-based government child and adolescent mental health service. Clients are children and young people experiencing moderate to severe mental health problems, along with their families or carers. Services include assessments, individual counselling, counselling and support for families, group programs for children and young people, and parent education.	No	Up to 17
**6**	Sydney, NSW	Free service based at a charitable organisation. Clients are families with young children who are displaying behaviour problems, including physical and verbal aggression, non-compliance, tantrums, destructive behaviour, hyperactivity, sibling rivalry, anxiety and withdrawal. The clinic works with families using the Parent-Child Interaction Therapy (PCIT) model. PCIT is based on play and aims to increase positive behaviours and reduce negative behaviours by strengthening the parent-child relationship and providing strategies for managing child behaviour. No self-referral.	No	15 months to 4 years
**7**	Melbourne, VIC	Free, hospital-based psychology service. Provides comprehensive assessment and feedback, parenting work, exposure therapy for children with anxiety, trauma work, and cognitive behavioural therapy. Clients are patients of the hospital and have a hospital-based consultant. No self-referral.	No	Up to 17
**8**	Sydney, NSW	A non-government organisation that provides health services to children who live in rural and remote NSW and have limited access to local services. Caters to children with non-acute developmental, behavioural, learning, emotional and mental health disorders. Families travel to Sydney and receive an intensive service while they stay on-site for the duration of treatment. The service is provided free of charge, however there are costs involved for accommodation and travel to the service. No self-referral.	N/A (clients stay on-site for duration of treatment)	4 to 17
**9**	Sydney, NSW	Free service based at a not-for-profit charity organisation. Provides early intervention for vulnerable/at-risk families, with referral from the Department of Family and Community Services. Delivers parenting programs such as Tuning into Kids, Triple P, Wrapped in Angels, Step into Work, My Family My Team, and Circle of Security. No self-referral.	No	0 to 7
**10**	Sydney, NSW	Free, university-based treatment, teaching, and research clinic specialising in treatments for children with behavioural and emotional problems. Provides parent management training to assist parents in managing their child's behavioural difficulties.	No	3 to 16

ACT: Australian Capital Territory; NSW: New South Wales; SA: South Australia; VIC: Victoria; WA: Western Australia.

### Procedure

The Human Research Ethics Committee (HREC) at the University of Sydney provided overall ethics approval for this study. Where applicable, approval was also obtained from local ethics committees overseeing individual services: South Eastern Sydney Local Health District HREC (NSW); South Western Sydney Local Health District Research and Ethics Office (NSW); Illawarra Shoalhaven Local Health District Research Support Office (NSW); Australian National University HREC (ACT); Royal Children’s Hospital HREC (VIC); Women’s and Children’s Health Network HREC (SA); Child and Adolescent Health Service HREC (WA). The terms of approval included a waiver of participant consent, as it was not feasible to contact all previous clients whose casefiles were included in the review. Services de-identified client data, so that datasets provided to the researchers did not include any information that could be used to identify clients. Services were asked to provide retrospective data from at least 100 casefiles; they began with the most recently closed cases and worked through earlier casefiles until a sufficient sample size was reached, which resulted in different casefile date ranges across services. The final sample size at each service was dependent on available staff time and resources, as well as the number of casefiles available. Services also provided information about the type of treatments and programs offered, cost (if any), referral process, age range of children attending the service, and whether the service offered sessions outside working hours (as holding sessions at convenient times has been identified as a key factor influencing father attendance [13]). This information is displayed in Table 1.

Each service identified an individual within the service who was familiar with the data and could undertake the data extraction and coding. This included administrative assistants, researchers, nurses, and students. The person who undertook this task received detailed instructions from researchers at the University of Sydney, along with a data dictionary specifying the variables of interest and the coding system for data entry. Data were entered into an SPSS or Excel file. Each service initially conducted a pilot review of approximately 20 cases, which was submitted for checking by researchers at the University of Sydney, prior to undertaking the casefile review for the entire sample. The complete dataset for each service was reviewed by the University of Sydney researchers and any inconsistencies were discussed for clarification with the service/individual who undertook the data extraction. The individuals who undertook the review were paid an hourly rate for their time. Casefile reviews at two services (8 and 10) were conducted by University of Sydney researchers. The summary measures described below were calculated by University of Sydney researchers using the raw datasets provided by each service.

### Measures

#### Average number of parent sessions

For each case, the total number of parent sessions was tallied. Sessions were only counted when at least one parent was required to attend (child-only sessions were not included, as parent attendance on these occasions, e.g. accompanying the child to the service, was unlikely to constitute involvement in the actual session). Cases with no parent sessions, or where number of parent sessions was unknown, were excluded. The *average number of parent sessions* per family was then calculated for each service.

This measure was not calculated for service 8, as number of parent sessions for each case was not recorded.

#### Percentage of families with an available father/mother

For each case, it was established whether there was a father (or male caregiver) who could possibly attend sessions, including fathers who had separated from the child’s mother but were involved in the child’s life and may have been able to attend. Similarly, it was established whether the mother was available to attend sessions (at two services, 8 and 10, mother availability was not recorded). Mother and father availability was coded by the individual reviewing the casefiles at each service, and was thus influenced by the information included in the casefiles along with reviewer judgement. For example, where children lived in a two-parent family with both mother and father, a reviewer would code that the mother and father were ‘available’ even if they did not attend sessions. Where parents were separated/divorced, mothers and fathers were coded as ‘available’ if they were involved in the child’s life (e.g., provided care for the child at least some of the time), or if another caregiver (e.g., a new partner) was in a caregiving role. Where separated/divorced mothers or father were rated as ‘not available’ they were either not involved in the child’s life, or they were not able to attend sessions for another reason (e.g., lived interstate). Mothers and fathers were also rated as ‘not available’ if the participating parent was a single parent and there was no second caregiver. As family structure was sometimes complex, with more than two caregivers per family, reviewer judgement was required, and reviewers often discussed coding of complex cases with University of Sydney researchers. The *percentage of families with an available father* and *percentage of families with an available mother* was then calculated for each service.

#### Percentage of total parent sessions attended by available fathers/mothers

For each case, the number of sessions attended by available fathers and mothers was tallied individually (sessions in which information about father/mother attendance was unknown were not included). The number of sessions attended by each available father/mother was divided by the total number of parent sessions for the family (minus any sessions where father/mother attendance was unknown), to calculate the percentage of total parent sessions they attended. The mean *percentage of total parent sessions attended by available fathers/mothers* was then calculated for each service.

This measure was not applicable to service 8, where families stayed at an on-site residential facility for the duration of the child’s treatment. Available parents were coded as either attending or not attending the service, as no information was provided in the casefiles regarding attendance at individual sessions.

#### Percentage of available fathers/mothers who attended at least one session

Based on the data collected for the above variables, calculations were conducted to determine the *percentage of available fathers/mothers who attended at least one session* for each service.

For service 8, this measure was based on attendance or non-attendance of available fathers/mothers at the service.

#### Rate of self-referral for fathers/mothers

For each case, the referral source for the family was noted. Six services (1–5, and 10) accepted families via self-referral, i.e. referral from the mother or father. Three of the services accepted self-referral only, and three accepted a combination of self-referral and other referral types. For each of these services, the number of referrals from fathers/mothers was divided by the total number of mother *or* father referrals at the service, to calculate the *rate of self-referral for fathers/mothers*.

### Statistical analyses

All statistical analyses were performed by researchers at the University of Sydney. Descriptive statistics were used to explore the main outcome measures relating to father and mother attendance and referral. Two independent samples t-tests were conducted in SPSS Statistics Version 22.0 (IBM) to assess whether there were significant differences between available fathers and mothers across participating services in: 1) percentage of total parent sessions attended; and 2) percentage who attended at least one session.

## Results

In total, data were extracted from 2128 casefiles (each casefile representing one family), across the 10 services. Data extracted from the casefile reviews at each service are summarised in Table 2. The number of casefiles reviewed ranged from *n* = 87 to *n* = 490; all casefiles were from a 10 ½ year period prior to and including May 2017. The average number of parent sessions ranged from 4.6 to 6.5 across most services, with one outlier (4) having an average of 29.7 parent sessions. Across services, the percentage of families with an available father was on average 74.6% (range 47.1% to 97.5%). The percentage of families with an available mother was on average 96.4% (range 92.0% to 100.0%).

**Table 2:** Summary data extracted from casefiles at participating services.

Service number	Date range of casefiles	Number of casefiles	Avg. number of parent sessions	Families with available father (%)	Families with available mother (%)	Parent attendance rates: total sessions (mean %)		Parent attendance rates: at least one session (%)		Parent referral rates ^a^ (%)	
						Available fathers	Available mothers	Available fathers	Available mothers	Available fathers	Available mothers
**1**	Jan 2013—Sep 2016	121	5.3	97.5	100.0	42.7	87.2	67.0	95.0	9.4	90.6
**2**	Jan 2016—Nov 2016	121^b^	4.6	47.1	93.4	45.5	94.4	63.2	97.3	17.1	82.9
**3**	May 2014 –May 2017	93^c^	6.5	65.6	94.6	50.4	97.8	68.4	100.0	14.8	85.2
**4**	Jun 2010—Apr 2016	276	29.7	69.9	97.8	45.1	93.7	88.1	99.6	16.5	83.5
**5**	Jan 2016 –Dec 2016	199^d^	5.2	67.6	95.0	49.7	90.6	70.8	94.5	9.1	90.9
**6**	Mar 2009 –May 2016	250	6.2	93.6	100.0	47.8	87.5	65.9	91.5	N/A	N/A
**7**	Feb 2012—Sept 2016	160	5.0	81.3	98.7	40.7	94.6	66.4	98.7	N/A	N/A
**8**	May 2007—Aug 2012	331	N/A^e^	64.9	unknown^f^	N/A^e^	N/A^e^	53.1	unknown^f^	N/A	N/A
**9**	Jul 2007—Dec 2016	87	6.3	73.6	92.0	39.7	96.8	55.0	98.7	N/A	N/A
**10**	Jan 2007—Dec 2014	490	5.4	84.8	unknown^f^	72.0	unknown^f^	86.0	unknown^f^	8.9	91.1

^*a*^Six services accepted families via self-referral.

^b^*n* = 35 self-referred.

^c^*n* = 27 self-referred.

^d^*n* = 22 self-referred.

^e^Parent attendance coded as either attending or not attending service (no information about individual session attendance).

^f^Not included in dataset.

The mean percentage of total parent sessions attended by available fathers ranged from 39.7% to 72.0% across services; available mothers attended total parent sessions at mean rates of 87.2% to 97.8%. An independent samples t-test showed that across services there was a statistically significant difference in the percentage of total parent sessions attended by fathers (M = 48.18, SD = 9.69) and mothers (M = 92.83, SD = 4.01; t (15) = -12.11, *p* < 0.001, two-tailed).

Of those families with a father present, the percentage of fathers who attended at least one parent session ranged from 53.1% to 88.1% across services; for available mothers, this figure ranged from 91.5% to 100.0%. An independent samples t-test revealed that across services there was a statistically significant difference in the percentage of fathers (M = 68.39, SD = 11.34) and mothers (M = 96.93, SD = 2.98; t (16) = -6.89, *p* < 0.001, two-tailed) who attended at least one parent session.

For services that accepted family self-referral, rates of father referral were on average 12.6% (range 8.9% to 17.1%); rates of mother referral were on average 87.4% (range 82.9% to 91.1%). As only six of the services accepted families via self-referral, and three of these included a small number of self-referred cases, it was deemed not appropriate to apply a test of statistical significance.

## Discussion

In the current study, father attendance rates at 10 Australian child mental health services were examined via retrospective casefile review. This method was chosen as none of the services routinely collected prospective data. Results revealed that the percentage of *total parent sessions* attended by available fathers was on average 48.2% (ranging from 39.7% to 72.0% across services). Perhaps unsurprisingly, the percentage of available fathers who attended *at least one session* was higher, at 68.4% on average (ranging from 53.1% to 88.1% across services).

Both measures of attendance demonstrated that fathers are attending sessions at significantly lower rates than mothers. Despite this finding, the results indicate that levels of father engagement in services may be higher than shown in previous studies. For example, across services over two thirds of available fathers attended at least one session, whereas previous reports have documented that only 20% of participants enrolling in parenting interventions are fathers [4]. Interestingly, attendance rates at different services were relatively consistent despite the fact that the percentage of families with an available father varied greatly across services (from 47.1% to 97.5%). This suggests that levels of father engagement remain constant even in areas where fewer families have a father available to participate in services. In terms of benchmarking rates of father involvement in interventions, this highlights the importance of considering whether there is a father available to attend, and raises the possibility that previous studies may have underestimated rates of father attendance by including families where there is no available father. The rates of father attendance in the present study should, however, be interpreted with caution, as the services that participated may have had a greater propensity towards father-inclusive practice than other services in the community, potentially resulting in selection bias. In addition, all 10 services were located in urban areas; although one Sydney-based service (8) caters to clients from rural and remote areas, these families stay on site for the duration of treatment. Therefore, the present sample is unlikely to include many rural families who may experience additional practical barriers to attendance, such as lengthy travel distances. In any case, while it appears that fathers are attending parenting interventions more frequently than expected, there is still clearly room to increase father engagement in services, particularly as some services are achieving higher rates of father attendance than others.

Overall, the majority of services had similar levels of father attendance (around 40–50% of total parent sessions; with around 60–70% of available fathers attending at least one session). It was not possible to analyse patterns in the data based on service, family or casefile characteristics due to the small number of services and the fact that data was not collected on some variables that may have influenced attendance rates (e.g., socio-demographics of families). However, there were a number of notable patterns in the data that may have been influenced by service, family and casefile characteristics. First, one service (10) had markedly high rates of father attendance, both in terms of proportion of total parent sessions (72.0%) and attending at least one session (86.0%). Given that this service is a university-based clinic that accepts families largely by self-referral (as opposed to referral from other services or government agencies), these fathers may simply be more motivated to attend sessions, although the low rate of father-instigated referral (8.9%) suggests this may not be the case. Since fathers are underrepresented at the initial referral or ‘Connecting’ stage, it seems more likely that they are being successfully engaged throughout the intervention (i.e. during the ‘Attending’ stage). Indeed, the high rates of father attendance may be associated with frequent use of father-inclusive practices, as the service routinely emphasises the importance of continued father involvement throughout intervention and problem-solving difficulties in relation to father attendance. Future research could explore rates of active father ‘Participation’ during sessions and ‘Enactment’ of strategies (e.g. homework completion), to fully examine father engagement according to the CAPE model of parental engagement [11]. It should also be noted that this service had the highest number of casefiles reviews, so this factor may have positively influenced the father engagement rates.

Second, the service with the lowest rates of father attendance (9) provides early intervention for at-risk families via referral from the Department of Family and Community Services, with no self-referral from parents. Thus, it may be that the lower rates of father engagement in this service were due to a greater number of vulnerable families when compared with the other participating services. It is nonetheless encouraging to note that the level of father attendance at this service is comparable to the other services included in this study, particularly as these parents may not be actively seeking help. Importantly, the lower rates of father attendance may have also been due to casefile characteristics, as this service also had the smallest number of casefiles reviewed, as well as the greatest date range for cases (cases from ~9 years were included).

Third, the service (4) that reported the highest rate of father attendance for at least one session (88.1%) was also the only service that offered sessions outside work hours. It should be noted that this service had by far the highest average number of parent sessions per family (29.7 sessions), increasing the likelihood that available fathers would have attended at least some sessions. Nevertheless, the high rate of fathers attending at least one session may reflect the fact that this service provides a specialised intervention (Multisystemic Therapy) which involves clinical staff visiting client homes for sessions, with many of these visits occurring outside work hours to help include both parents in the intervention. This highlights the importance of service-level factors, such as offering sessions outside work hours or at a convenient location, that can facilitate at least some level of father engagement in interventions. Indeed, surveys of practitioners [14] and fathers in the community [13, 15] have identified convenient time and location as key factors in promoting father attendance at services or treatment for child behaviour.

Rates of father referral were low across all services that accepted families by self-referral, ranging from 8.9% to 17.1% (as compared to 82.9% to 91.1% for mother referrals). While this clearly indicates that fathers are not usually the instigators of help-seeking for their child, the reasons for this are less certain. It could be related to maternal ‘gatekeeping’ (e.g., [16]), whereby mothers’ beliefs about the paternal role may influence the level of father involvement in their child’s life. Focus group research with fathers has identified both maternal gatekeeping and stigma around help-seeking as barriers to fathers attending parenting interventions [17], and these factors may therefore play a key role during the family’s initial point of contact with services. It is also likely that service-level policies, such as offering sessions during work hours only, might not only act as practical barriers to father attendance at sessions, but similarly influence rates of father-instigated referrals. Further research is needed in relation to the apparently low rates of father involvement at this ‘Connecting’ stage of parental engagement, particularly as the present data is based on only six services that accepted self-referral, and three of these included just a small sub-sample of self-referred cases.

While it appears that the services we have sampled here are getting fathers ‘in the door’ (as evidenced by an average of 68.4% of available fathers attending at least one session), most are not maintaining high rates of attendance across all sessions. Given the importance of father participation for optimising intervention outcomes, this may compromise the quality of child mental health services currently delivered in Australia. The evidence from survey research suggests that many fathers in the community are willing to attend parenting programs [14]. However, there are various existing barriers to father engagement in services, particularly in relation to time, location and work commitments [6, 13, 15], therefore it is critical for practitioners and services to implement strategies that will afford available fathers every opportunity to attend. At the same time, it is important to note that it may not always be in the best interests of the child to engage both parents in the intervention. There may be circumstances in which the appropriateness of engaging the father (or the mother) should be further explored with the referring parent, such as when there are concerns related to domestic or family violence, drug and alcohol abuse, or antisocial or criminal behaviour.

The present benchmark rates will be particularly useful for measuring the impact of future initiatives to increase father engagement in services, implemented both at the individual practitioner and service level. In order to measure whether such changes are influencing father engagement, it is important that services continue to record and track both father and mother session attendance. This is a relatively simple and cost-effective way to ensure that rates of attendance are monitored over time, allowing services to evaluate whether service-level policies and practices intended to improve father engagement (e.g. providing sessions outside working hours, specific training programs in father engagement, or promotion of services to fathers) are impacting on rates of father attendance. Recording and analysing father and mother attendance data separately also allows for examination of research questions such as whether certain factors may differentially affect mother and father attendance in parenting interventions [6]. For example, provision of sessions outside work hours has been identified in previous research as a factor that could increase father attendance [13], however it is unclear how this would impact on mother attendance rates.

Although this study has several implications for understanding father engagement, there are some limitations. First, it is unclear the extent to which the services included here are representative of all Australian child mental health services. Some Australian states and territories were not represented (namely Queensland, Northern Territory and Tasmania) and we were not able to include services based in regional or remote areas. While we aimed to recruit a broad range of child mental health services, it is possible that the services that participated may have a particular focus on father inclusion, and rates of father engagement in these services may therefore be higher than others in the community. To overcome this limitation, future research could randomly select services to report on father attendance, or include a larger number of services. Second, we relied on data extraction from casefile notes to provide the data for this study, and for some services, data were missing from some variables, which may have reduced the validity of the findings. In addition, as different individuals undertook the casefile review at different services, and inter-rater reliability could not be examined, it was not possible to ascertain the reliability of the data. Third, the date range of the casefiles varied widely across services, which may have introduced bias into the measurement of attendance rates at some sites. Individual casefile dates were not recorded in this study, so we were also unable to examine whether there was any change in father attendance rates at individual services over time. This highlights the importance of service-level tracking of father (and mother) session attendance across time, as well as the need for future benchmarking studies to include dates of intervention for individual cases. Finally, it was not possible within the scope of this study to examine variables that may have systematically impacted on levels of father attendance, such as service-level factors (e.g. service type, service location, use of father-inclusive policies or practices), family factors (e.g. socioeconomic status, relationship of father/male caregiver to child) or casefile factors (e.g. number of cases, date range of casefiles). To strengthen the findings of future research, it would be useful to measure and include these variables in order to examine moderators of father attendance rates.

Despite these limitations, a major strength of this research is that it provides data on rates of father attendance from 10 child mental health services across five Australian states and territories, including casefiles from more than 2000 families. Thus, in this study we have provided the first baseline of father attendance against which to measure future initiatives designed to improve father engagement.
